# IFN-γ Production to Leishmania Antigen Supplements the Leishmania Skin Test in Identifying Exposure to *L. braziliensis* Infection

**DOI:** 10.1371/journal.pntd.0001947

**Published:** 2012-12-20

**Authors:** Daniel Schnorr, Aline C. Muniz, Sara Passos, Luiz H. Guimaraes, Ednaldo L. Lago, Olívia Bacellar, Marshall J. Glesby, Edgar M. Carvalho

**Affiliations:** 1 Serviço de Imunologia, Complexo Hospitalar Universitário Prof. Edgard Santos, Universidade Federal da Bahia, Salvador, Brazil; 2 Columbia University College of Physicians and Surgeons, New York, New York, United States of America; 3 Instituto Nacional de Ciência e Tecnologia de Doenças Tropicais (CNPq/MCT), Salvador, Brazil; 4 Division of Infectious Diseases, Department of Medicine, Weill Cornell Medical College, New York, New York, United States of America; National Institutes of Health, United States of America

## Abstract

**Background:**

Cutaneous leishmaniasis due to *L. braziliensis* (CL) is characterized by a positive delayed type hypersensitivity test (DTH) leishmania skin test (LST) and high IFN-γ production to soluble leishmania antigen (SLA). The LST is used for diagnosis of CL and for identification of individuals exposed to leishmania infection but without disease. The main aim of the present study was to identify markers of exposure to *L. braziliensis* infection.

**Methodolgy/Principal Findings:**

This cohort study enrolled 308 household contacts (HC) of 76 CL index cases. HC had no active or past history of leishmaniasis. For the present cross-sectional study cytokines and chemokines were determined in supernatants of whole blood culture stimulated with SLA. Of the 308 HC, 36 (11.7%) had a positive LST but in these IFN-γ was only detected in 22 (61.1%). Moreover of the 40 HC with evidence of IFN-γ production only 22 (55%) had a positive LST. A total of 54 (17.5%) of 308 HC had specific immune response to SLA. Only a moderate agreement (Kappa = 0.52; 95% CI: 0.36–0.66) was found between LST and IFN-γ production. Moreover while enhancement of CXCL10 in cultures stimulated with SLA was observed in HC with DTH+ and IFN-γ+ and in patients with IFN-γ^+^ and DTH^−^, no enhancement of this chemokine was observed in supernatants of cells of HC with DTH^+^ and IFN-γ^−^.

**Conclusions/Significance:**

This study shows that in addition of LST, the evaluation of antigen specific IFN-γ production should be performed to determine evidence of exposure to leishmania infection. Moreover it suggests that in some HC production of IFN-γ and CXCL10 are performed by cells not involved with DTH reaction.

## Author Summary

Both control of *L. braziliensis* infection and development of cutaneous leishmaniasis (CL) are dependent on the host immunological response. Due to the difficulty of finding parasites in leishmanial lesions, a delayed type hypersensitivity reaction - leishmania skin test (LST), is widely used to diagnose CL. In areas of *L. braziliensis* transmission a positive LST is also documented in up to 18% of individuals without disease, who are considered to be putatively resistant to leishmania infection. However the mechanisms involved in the control of parasite grow is not known. The aim of this study is to identify tests that could determine in house contact of CL (HC) without past or current evidence of leishmaniasis exposure to leishmania infection. We found that of the 308 HC, 36 (11.7%) had a positive LST but in these IFN-γ was only detected in 22 (61.1%). Moreover of the 40 HC with evidence of IFN-γ production only 22 (55%) had a positive LST. Therefore at least the two tests, the LST and IFN-γ production, should be used to determine exposure to *L. braziliensis*. Identification of subjects exposed to leishmania infection that may or may not develop CL is highly relevant to understand pathogenesis of *L. braziliensis* infection.

## Introduction

American tegumentary leishmaniasis (ATL) is caused predominantly by *Leishmania braziliensis*, *L. guaynensis*, *L. mexicana* and *L. amazonensis* [1], [2]. It is endemic in South and Central America and cutaneous leishmaniasis (CL), characterized by well delimited ulcers with raised borders, is the most common clinical picture of ATL. The main characteristics of the immunological response in CL are a strong Th1 type immune response to soluble leishmania antigen (SLA), demonstrated by positive delayed type hypersensitivity (DTH) reaction to the leishmania skin test (LST), as well as lymphocyte proliferation and production of high levels of IFN-γ and TNF-α [3], [4]. Only a few parasites are found in the lesions due to *L.braziliensis* and because of this the leishmania skin test (LST) is widely used for diagnosis of ATL. A positive test in a patient with a typical cutaneous lesion has a high predictive value [5], [6]. The LST has been also used to measure exposure to leishmania infection, and a positive LST in the absence of clinical manifestations of ATL has been documented in up to 17% of healthy individuals living in endemic areas of *L. braziliensis* [6]. Individuals with a positive LST who do not develop leishmaniasis are considered as having a subclinical *L. braziliensis* infection [7]. Although a concordance between DTH and *in vitro* tests of cell mediated immune response is expected, discordant results between IFN-γ production and tuberculin skin test (TST) have been shown in individuals with latent tuberculosis [8]–[10]. Therefore it is important to determine whether this discordance also occurs in subclinical *L. braziliensis* infection as well as to evaluate if other tests may be indicative of exposure to leishmania infection.

As the ratio from infection to disease based on LST is 3.7 to 1, about 25% of individuals who are exposed to *L. braziliensis* will develop cutaneous leishmaniasis [7]. It is known that early events after penetration of leishmania in the skin are important to determine the outcome of leishmaniasis. Therefore characterization of immune response early after the infection is highly relevant. Moreover, an early detection of individuals exposed to *L. braziliensis* will allow a comparative analysis between individuals who will develop or not develop disease. The aim of this study was to establish and follow a prospective cohort of household contacts of CL patients, to evaluate initially markers of exposure to leishmania infection and ultimately to identify markers that are associated with resistance or susceptibility to develop disease. Our initial evaluation of this cohort indicates based in a cross-sectional study that more than one test is needed to determine exposure to *L. braziliensis*. In addition to LST, IFN-γ production in SLA stimulated cultures should be determined. Moreover, the production of CXCL10, a chemokine associated with recruitment and activation of T cells, gives support that in some cases CXCL10 and IFN-γ are produced by cells not involved with DTH.

## Materials and Methods

### Ethics statement

This study was approved by the Ethical Committee of the Federal University of Bahia. Written informed consent was obtained from all enrolled subjects.

### Study site

This study was conducted in Corte de Pedra, a rural region in Northeastern Brazil endemic for ATL, where we have performed clinical and immunological studies for over 25 years [3], [11], [12]. The area was previously dominated by Atlantic rainforest and is now a mostly deforested agricultural community. *Lutzomyia whitmani* and *Lu. intermedia* sandflies that transmit *L. braziliensis* are endemic in the local fauna [2], [13]. The health post of Corte de Pedra was created in 1986 and is a reference center for diagnosis and treatment of CL and is staffed by medical personnel from the Federal University of Bahia.

### Study population and study design

This is a cohort study enrolling household contacts (HC) of patients with history of CL. Index cases (IC, N = 76) were recruited at the Corte de Pedra health post. An index case was defined as a patient with confirmed CL diagnosed at the Corte de Pedra health post within two years prior to enrollment in the study, living within a 10 km radius of the health post. Patients with evidence of mucosal or disseminated leishmaniasis were not considered for enrollment as index cases. Researchers visited index cases in their homes to recruit HC from January to April 2010. Household contacts (N = 533) were defined as individuals without history of any type of leishmania infection who were living in the same home as the index case at the time of enrollment in the study and at the time of diagnosis of CL by the index case. Cutaneous leishmaniasis (CL) patients were diagnosed based on a typical clinical leishmaniasis lesion, associated with a positive leishmania skin test (LST) and documentation of parasites in culture or by histopathology. After obtaining informed consent, a negative history of CL in HC was established by a medical interview, assessing for symptoms consistent with previous CL or ML infection, and negative physical exam looking for scars consistent with past CL or ML on the skin, nose and soft palate. Exclusion criteria for HC were age less than 2 or more than 65 years, or frequent stays outside of the endemic area. After the initial immunologic studies a cross-sectional study was performed first comparing epidemiological and chemokine data among HC with evidence or without evidence of immune response to soluble leishmania antigen (SLA). Second, comparing cytokine data among groups according evidence of IFN-γ production and response to LST.

### *Leishmania* antigen

SLA for the skin test was prepared with an isolate of *L. braziliensis* as previously described [14]. Briefly, promastigotes of *L.braziliensis* were grown in Schneider's medium supplemented with 10% fetal bovine serum and 2% human urine. The promastigotes were washed in sterile phosphate buffered saline (PBS), resuspended in lysis solution (Tris HCl, EDTA and leupeptin), immersed in liquid nitrogen, and thawed at 37°C. After freeze-thaw procedure, they were sonicated. The disrupted parasites were centrifuged at 14,000G and assayed for total protein (BCA Protein Assay Kit, Thermo Scientific). For *in vitro* testing the filtrate was adjusted to a concentration of 500 µg/mL with sterile PBS. For the LST, the filtrate was adjusted to a concentration of 250 µg/mL with sterile PBS containing Tween 80 and phenol at final concentrations of 0.0005%(w/v) and 0.28% (w/v) respectively.

### Evaluation of IFN-γ and chemokine production

Once a negative history of CL was established, heparinized peripheral blood (10 mL) was collected immediately before LST was performed. About 6 h after collection, 1 mL of whole blood aliquots was dispensed into a 24-well tissue plate. SLA at 20 µg/mL, 50 µl of phytohemaglutinin (GIBCO, Grand Island- NY) as positive control or medium (negative control) were added to each well and incubated at 37°C 5% CO2 for 72 hours. Plasma supernatants (300–400 uL) were collected, and samples were stored at −20°C. The levels of IFN-γ released were quantified by ELISA, using commercially available reagent (BD OpTEIA, San Diego-CA). A standard curve was used to express the results in pg/ml. A positive test was defined as any detectable IFN-γ level after subtracting the SLA stimulated IFN-γ levels by unstimulated IFN-γ levels.

The levels of CXCL9, CXCL10, and CCL2 were measured by ELISA using commercially available reagents (BD OpTEIA, San Diego-CA). A standard curve was used to express the results in pg/ml. Chemokine data are summarized separately as spontaneous production (medium) and SLA-induced production (Ag).

### Leishmania skin test

The LST was performed after collection of blood for the *in vitro* test to avoid the possible influence of the skin test reaction on the immune response determined *in vitro*. SLA for the skin test was prepared with an isolate of *L. braziliensis* as previously described [14]. For LST, 0.1 mL (25 µg/mL) of the SLA was injected intracutaneously on the volar surface of the forearm, and the greater diameter of induration was measured 48–72 h later. Induration of ≥5 mm was defined as a positive reaction.

HC were considered to have been exposed to leishmania if they demonstrated a positive immune response to *L. braziliensis* antigen in either the *in vitro* and *in vivo* test.

### Statistical analysis

The analysis of the concordance between LST and IFN-γ production was performed by calculating a κ statistic for agreement with 95% confidence interval. Demographic characteristics were compared across subject groups as follows: For continuous variables, one-way ANOVA or Kruskal-Wallis tests were used and if the overall P-value was <0.05, pair-wise Bonferonni or Dunn's post-hoc tests were performed. For categorical variables, chi-square or Fisher's exact test were performed. Student T test was used to compare the means of variables following normal distribution. For analysis of IFN-γ and chemokines production in unstimulated and stimulated culture, the Wilcoxon rank-sum test was used. Correlations were performed by the method of Spearman. STATA version 11 (College Station, TX) and GraphPad InStat3 (La Jolla, CA) statistical software were used for all the analyses.

## Results

**Figure 1:**
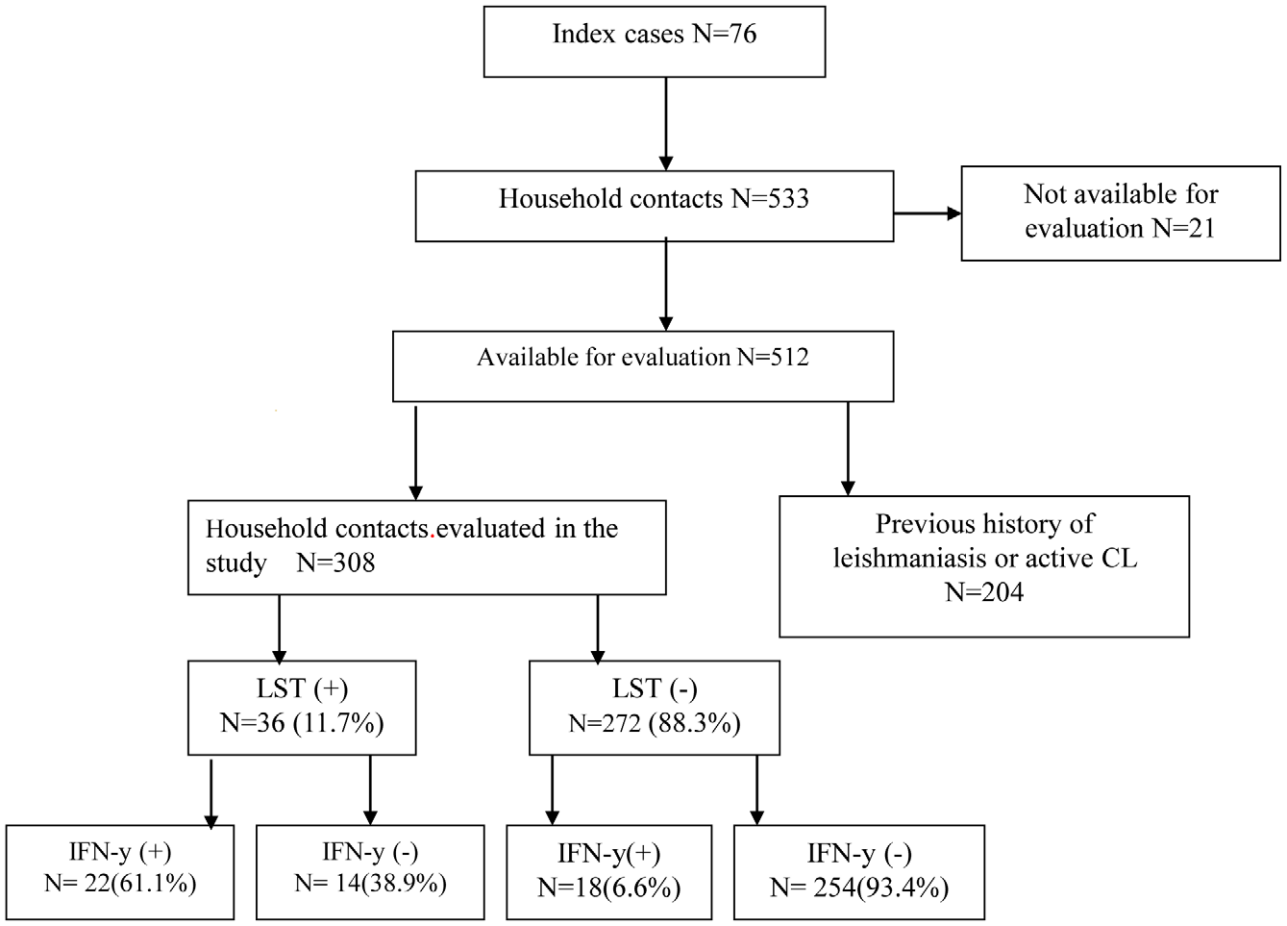
Establishment of a cohort of HC of CL patients in *L. braziliensis* endemic area.

Figure 1 depicts the design of the cohort study and distribution of study subjects. A positive LST was observed in 36 (11.7%) of the 308 HC tested and IFN-γ production was detected in 40 HC (12.9%). However in the 36 individuals with positive LST, production of IFN-γ was only observed in 22 (61.1%), and in the 40 HC with evidence of production of IFN-γ, only 22 (55%) had a positive LST. Considering evidence of immunological response to leishmania antigen as a positive LST and/or IFN-γ production in cultures stimulated with SLA, 54 (17.5%) of 308 HC had specific immunological responses to SLA. IFN-γ production was documented in supernatants from cells of all HC stimulated with phitohemaglutinin.

The demographic and epidemiological aspects of index cases and HC with and without evidence of immune response to SLA are shown in Table 1. There was no difference among the index cases and HC with evidence of immune response in all variables analyzed (age, gender, occupation, years living in the area as well as in the same house). There was also no difference among the three groups regarding the time of arriving at home. However, differences were found between HC with and without evidence of exposure to leishmania infection regarding age, occupation, time living in the endemic area and time living in the same house. Household contacts without evidence of immune response were younger and the majority was students; consequently, they had less time living in the same house and in the same endemic area of index cases than HC with evidence of immune response.

**Table 1:** Demographic and epidemiologic features of patients with cutaneous leishmaniasis and household contacts with and without evidence of immune response to leishmania antigen.

Variables	Index Cases (N = 76)	Household Contacts with Evidence of Immune Response (N = 54)	Household Contacts without Evidence of Immune Response (N = 254)	Overall P-value
Age (years)	22.7+/−15.0^a^	23.1+/−15.5^b^	16.8+/−11.7^a^^, ^ b	0.001*
Gender (% male)	41 (54.0)	28 (51.8)	123 (48.4)	0.67 **
Occupation, N (%)				
- Agriculture	17 (22.4)	10 (18.5)	52 (20.5)	0.058 **
- Domestic	27 (35.5)	19 (35.2)	56 (22.0)	
- Student/Other	32 (42.1)	25 (46.3)	146 (57.5)	
Years in Endemic Area	21.7+/−14.8^c^	22.7+/−15.0^d^	16.4+/−11.4^c^^, ^ d	0.002*
Years in same house	12.7+/−11.4^e^	14.6+/−14.3^f^	9.1+/−6.9^e^^,^ f	0.001*
Arriving home, N (%) ***	63 (82.9)	45 (83.3)	208 (81.9)	0.96 **

Values with identical superscripts are significantly different. Statistical tests and p-values are below.

P-values for pair-wise Bonferroni post-hoc tests:

a = 0.002,

b = 0.004,

c = 0.005,

d = 0.003,

e = 0.009,

f <0.001.

* one-way ANOVA test.

** Pearson's chi-square test.

*** before 4:00 pm.

In order to determine the best test to detect exposure to leishmania infection, we compared the ability of the LST and IFN-γ production to identify exposure to *L. braziliensis*. When assessing the concordance between LST and IFN-γ production, among those who had at least one test positive, 61.1% of the HC were positive for both tests and there was moderate agreement beyond that expected by chance (Kappa = 0.49; 95% CI: 0.34–0.64). When the two tests were analyzed in the whole population a moderate concordance between IFN-γ and LST (Kappa = 0.52; 95% CI: 0.36–0.66) was confirmed.

As chemokines are produced early in leishmania infection and they participate in the immune response by activation and recruitment of T cells, expression of chemokines related to lymphocyte and monocyte recruitment were determined in all individuals with evidence of immune response (N = 54) and in a sub-group (N = 51) of HC without evidence of immune response. These 51 HC without evidence of immune response were selected among individuals living in the same house of HC with evidence of immune response. The IFN- γ, CXCL9, CXCL10 and CCL2 levels in IC, HC with evidence of immune response and HC without evidence of immune response are shown in Table 2. As expected IFN-γ levels were higher in IC than in the other groups. High levels of CXCL9 and CCL2 were found in unstimulated cultures of both IC and HC with and without evidence of immune response. While there was no difference regarding the production of CCL2 in unstimulated culture, the levels of this chemokine were higher in HC with evidence of immune response in cultures stimulated with SLA when compared with the control group (p<0.001). With regard to CXCL9 and CXCL10, levels of SLA induced chemokines were higher among the IC and HC with evidence of immune response than the levels observed in the control group (P<0.05).

**Table 2:** Spontaneous and SLA induced IFN-γ and chemokines production by cells of household contacts of cutaneous leishmaniasis patients with and without evidence of immune response to leishmania antigen.

	Index cases (N = 20)	Household contacts with evidence of immune response to SLA (N = 54)	Household contacts without evidence of immune response to SLA (N = 51)	p-value
Age	22.7+/−14.7	23.4+/−15.5	22.8+/−16.1	p>0.05*
Gender (% Male)	10 (52.6)	28 (51.8)	24 (47.0)	p = 0.367^†^
IFN-γ (Medium)***	0 (0;0)	0 (0;0)	0 (0;0)	
IFN-γ (Ag)***	1189 (830;1502)^a^^;^ b	78 (4;1338)^a^^;^ c	0(0;0)^b^^;^ c	P<0.0001**
CXCL9 (Medium)***	4549 (2099;9313)^d^	2528 (1588; 5822)^e^	923 (456; 2569)^d^^;^ e	p<0.0001**
CXCL9 (Ag)***	11843 (10323; 12882)^f^	9190 (2744; 54675)^g^	1316 (580; 3243)^f^^;^ g	p<0.0001**
CXCL10 (Medium)***	3385 (1454; 4876)^h^^;^ i	0 (0; 317)^h^^;^ j	290 (0; 645)^i^^;^ j	p<0.0001**
CXCL10 (Ag)***	32404 (26695; 37370)^k^^;^ l	1376 (0; 28259)^k^	621 (193; 1894)^l^	p<0.0001**
CCL2 (Medium)***	14240 (8530;22590)	11000 (6440; 22920)	11220 (4920; 28960)	p = 0.84**
CCL2 (Ag)***	59900 (46460;73430)^m^^;^ n	30550 (15900; 38540)^m^	12620 (5400; 28720)^n^	p<0.0001**

Values with identical super-scripts are significantly different. Statistical tests and p-values are below:

P-values for Kruskal Wallis Dunn's post test:

a <0.05,

b <0.001,

c <0.001,

d <0.001,

e <0.001,

f <0.001,

g <0.001,

h <0.001,

i = <0.001,

j = 0.01,

k <0.001,

l <0.001,

m <0.001,

n <0.001.

* one-way ANOVA test;

** Kruskal Wallis test;

*** Results expressed as Median value (inter-quartile range).

**Figure 2:**
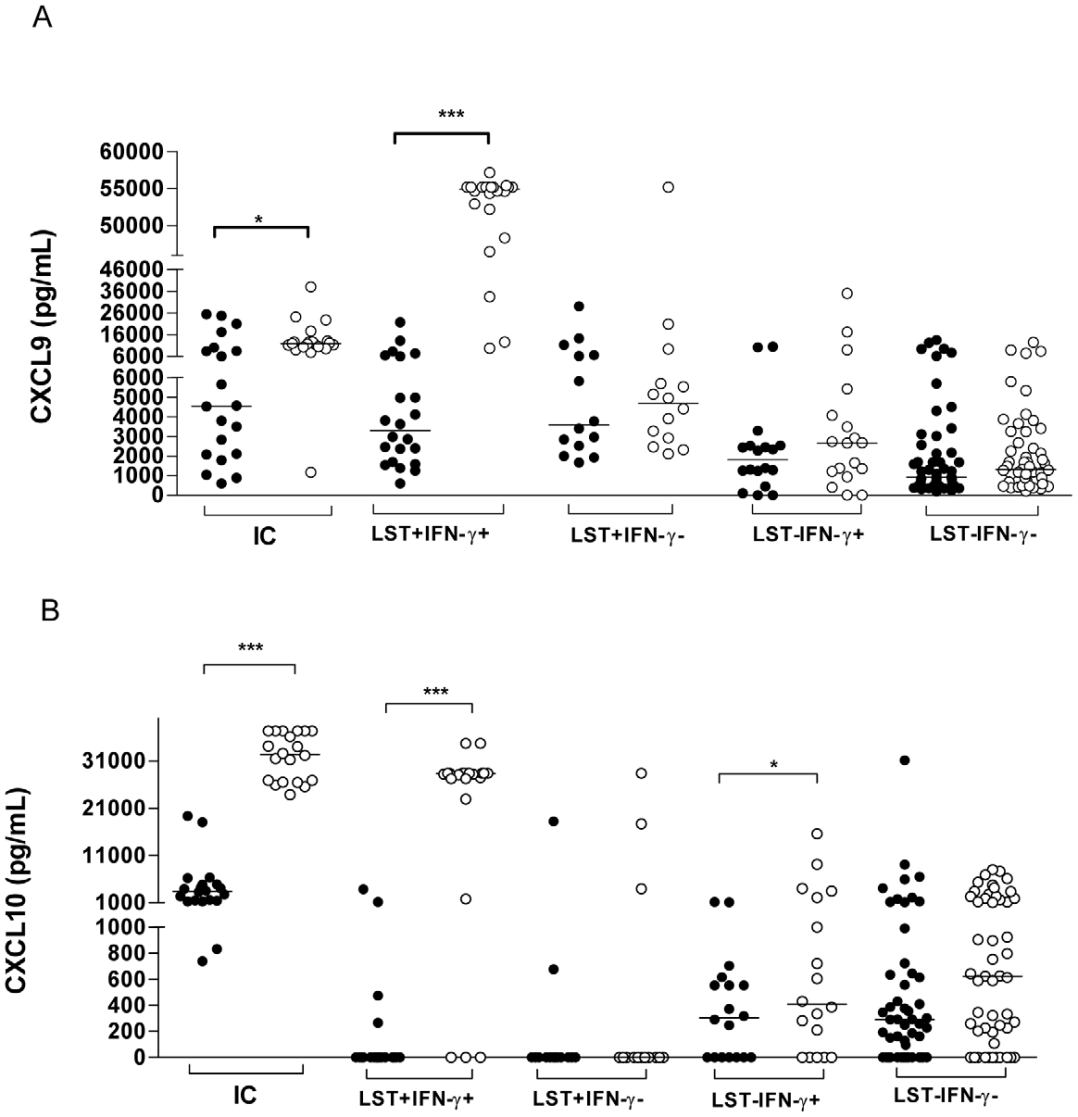
Production of CXCL9 and CXCL10 in cultures with or without stimulation with soluble *Leishmania* antigen. Aliquots of 1 ml of heparinized peripheral blood were cultured in the absence (medium) or stimulated with SLA for 72 hours. Chemokines production was determined by ELISA and results are expressed as pg/ml. Figure A shows the data obtained for CXCL9 and Figure B CXCL10. Values statistically different are indicated as: *** p<0.0001; *p<0.05; Wilcoxon test; GraphPad Prism 4. Medium; SLA.

**Figure 3:**
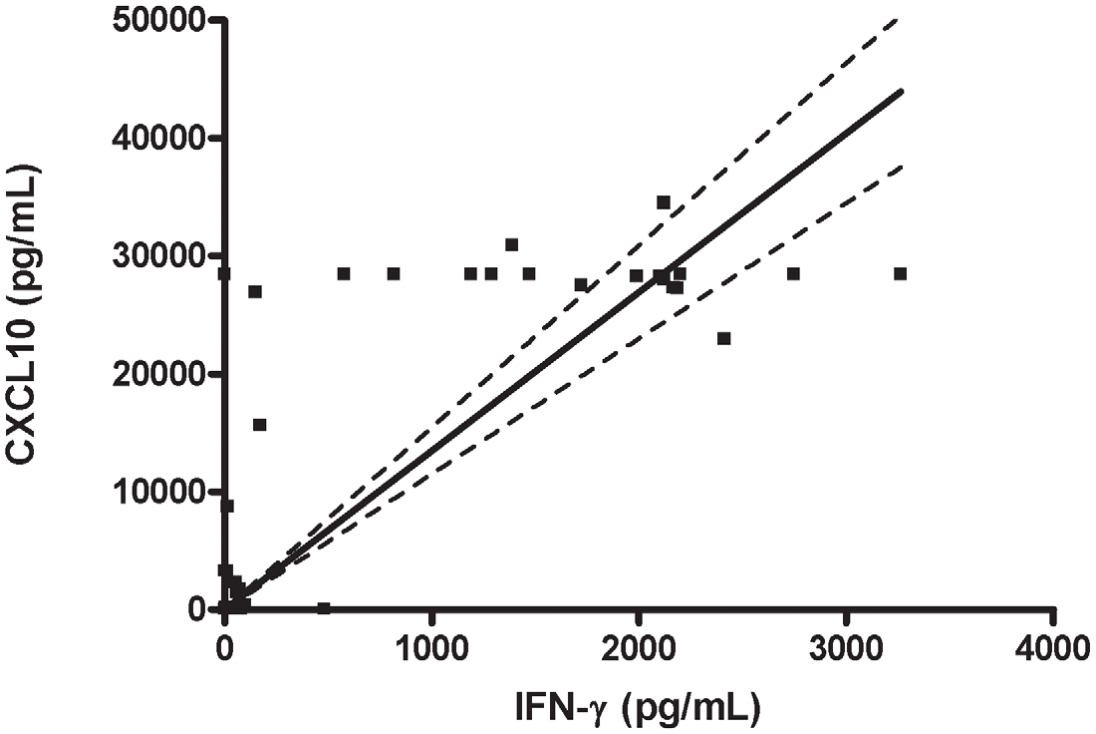
Correlation between IFN-γ and CXCL10 production to soluble *Leishmania* antigen. The data was obtained from 54 household contacts (HC) with evidence of immune response and 51 household contacts without evidence of immune response which were selected among individuals living in the same households as HC with evidence of immune response. Values of IFN-γ (pg/ml) and CXCL10 (pg/ml) from 105 HC were plotted in Figure 3 and these data were analyzed by the Spearman correlation test. P<0.0001, r = 0,74.

To evaluate if the chemokine production was associated with a positive LST or ability to produce IFN-γ *in vitro* upon SLA stimulation, the 105 HC who had chemokines determined were divided into 4 sub-groups: 1) LST positive and evidence of IFN-γ production (LST+ IFN-γ+); 2) LST+ and absence of IFN-γ production (LST+ IFN-γ−); 3) LST negative and IFN-γ positive (LST− IFN-γ+); 4) Both LST and IFN-γ negative (DTH− LST−). The production of CXCL9 and CXCL10 in both spontaneous and in SLA stimulated cultures is shown in Figure 2. The levels of CCL2 were increased in cultures stimulated with SLA in all individuals who had a positive LST or IFN-γ production as well as in HC without evidence of immune response (data not shown). However, the high levels of CCL2 in cultures of individuals without evidence of immune response prevent the use of CCL2 as an indicator of evidence of immune response to SLA. An increase in the production of CXCL9 in cultures stimulated with SLA was documented in IC and HC with evidence of IFN-γ production and with positive LST (Figure 2A). In the majority of HC, positivity in both tests (LST and production of IFN-γ) was required for a high production of CXCL9 in SLA stimulated cultures. Production of CXCL10 in unstimulated and SLA stimulated cultures is shown on Figure 2B. IC and subjects with positive LST and IFN-γ production, as well as those who were LST negative but produced IFN-γ, had increased CXCL10 in SLA stimulated cultures. However, one striking observation was that while CXCL10 production was detected in SLA stimulated cultures of HC with a negative LST but producers of IFN-γ, absence or very low production of CXCL10 was observed in SLA stimulated cultures of individuals who, despite having a positive DTH, had an absence of IFN-γ production to SLA. Moreover, there was a positive correlation between production of IFN-γ and CXCL10 in SLA stimulated culture (Figure 3).

## Discussion

Epidemiologic and clinical studies of ATL have focused on determining the influence of host, parasite and environmental factors on the development of the different clinical forms of leishmaniasis [15], [7], [11], [16]. However, there is a lack of studies in individuals who have evidence of exposure to leishmania but may or may not develop disease. The documentation of a positive LST in the absence of current or past history of CL is the only test that has been used to identify individuals who have a subclinical form of *L. braziliensis* infection [6], [7]. Herein we showed that the use of *in vitro* immunologic tests such as production of IFN-γ not only increases the number of individuals with evidence of exposure to leishmania infection, but we also demonstrate discordant results between LST and *in vitro* IFN-γ production by cells of HC. Moreover, the increase in CXCL10 production in SLA stimulated cultures was predominantly associated with IFN-γ production rather with a positive LST.

The primary objective of this study was to identify individuals recently exposed to leishmania infection. A limitation of this type of study is that we cannot be sure when exposures to leishmania have occurred. However, the comparative analysis of demographic characteristics of the index cases and of the HC with and without evidence of immune response suggested that HC with evidence of immune responses more closely resembled index cases, supporting the contention that exposure to leishmania infection in this population likely occurred close to the period that the CL index cases acquired the parasite.

The DTH and *in vitro* immunologic response determined by lymphocyte proliferation or cytokine production to recall antigens have been widely used to determine evidence of cell mediated immune response [17]–[20], [9]. In patients with CL as well as in patients with mucosal leishmaniasis due to *L. braziliensis* infection there is a strong association between the positivity of LST and production of IFN-γ as well as evidence of lymphocyte proliferative response to SLA [3], [17], [15]. Because of the high predictive value of LST in the diagnosis of CL, this test has also been used to identify exposure to leishmania infection among healthy individuals living in areas of *Leishmania* sp transmission [21], [6], [7]. Individuals with a positive LST and absence of current or past history of leishmaniasis in areas of *L. braziliensis* transmission are considered as having subclinical *L. braziliensis* infection. We have previously shown that these individuals with a positive LST have a lower production of IFN-γ in SLA stimulated cultures than patients with CL, and in some of them even no detectable IFN-γ levels were found in supernatants of lymphocytes stimulated with SLA [7], [16]. In the present study in addition to showing that HC that have a positive LST may not produce IFN-γ upon SLA stimulation, there were also HC who produced IFN-γ but had a negative LST. Discordance between DTH and IFN-γ production has been observed in subjects with latent tuberculosis [22], [8], [9], [10], and several factors may explain the discordance between LST and *in vitro* IFN-γ production: 1) Presence of suppressor factors *in vivo* that prevent the documentation of DTH; 2) Production of IFN-γ by non T cells; 3) Lack of effector or effector memory T cells but presence of memory T cells. Discordance between DTH and *in vitro* tests have been documented in patients with active tuberculosis as well as in individuals with subclinical *L. chagasi* infection [9], [23]. In such cases malnutrition as well as the presence of soluble suppressor factors may explain the absence of response *in vivo* but the occurrence of response *in vitro*. Usually in this case restoration of the DTH test occurs after specific therapy as well as with improvement in nutritional status [24], [25]. Regarding memory, studies in an experimental model of leishmaniasis have shown that after control of leishmania infection memory effector cells may not be found but animals remain with central memory T cells [26]. IFN-γ in patient with CL is predominantly secreted by effector T cells or memory effector T cells [27]. However, the disappearance of these cells from the peripheral blood may make the *in vitro* test become negative. Since in DTH tests antigens are inoculated intradermally and immune response is evaluated 48 to 72 hours after the test, there is time for memory effector T cells that remain in lymph nodes or in other tissues to migrate to the site of the injection of SLA and react *in vivo* to leishmania antigen. As the participants of this study were healthy, well-nourished, and likely recently exposed to *L. braziliensis* based on the epidemiologic data, the more likely explanation for the discordance between the LST and IFN-γ production in the LST negative IFN-γ positive subjects is the production of IFN-γ by cells not involved with DTH. Giving support to this hypothesis is our data that CXCL10 was produced in HC with evidence of IFN-γ production but not in those with only a positive LST. Different cell types such as neutrophils, NK cells and NKT cells may produce IFN-γ [28]–[31] and future studies will address this subject.

This study shows that in *L. braziliensis* infection in addition to the LST, the documentation of exposure to leishmania antigen should be also evaluated by antigen specific IFN-γ production as it increases the evidence of exposure to leishmania infection from the 11.7% (as documented by LST) to 17%, when LST or IFN-γ were positive. The discordance between IFN-γ and LST was highlighted by the observation that production of CXCL10 was associated with IFN-γ production but not with a positive LST.

## Supporting Information

Checklist S1 **STROBE Statement.**
(DOC)
